# Unpacking mentalizing: The roles of age and executive functioning in self-other appraisal and perspective taking

**DOI:** 10.1177/17470218241311415

**Published:** 2025-01-08

**Authors:** Elena Poznyak, Lucien Rochat, Deborah Badoud, Ben Meuleman, Martin Debbané

**Affiliations:** 1 Developmental Clinical Psychology Research Unit, Faculty of Psychology and Educational Sciences, University of Geneva, Geneva, Switzerland; 2 Specialized Facility in Behavioral Addiction ReConnecte, Department of Mental Health and Psychiatry, University Hospitals of Geneva, Geneva, Switzerland; 3 Faculty of Psychology, UniDistance Suisse, Brig, Switzerland; 4 Swiss Center for Affective Sciences, University of Geneva, Geneva, Switzerland; 5 Faculty of Psychology and Educational Sciences, University of Geneva, Geneva, Switzerland; 6 Research Department of Clinical, Educational and Health Psychology, University College London, London, UK

**Keywords:** Self-other control, mentalizing, adolescence, trait attribution, self-other appraisal, perspective taking

## Abstract

Mentalizing involves a number of psychological processes designed to appraise self and others from different points of view. Factors affecting the flexibility in the ability to switch between self-other representations and perspectives remain yet unclear. In this study, we sought to (a) assess individual variability in processing and switching between self and other-oriented mental representations and perspectives in a sample of typically developing youths and (b) examine how age and executive functioning may affect this switching process. A total of 88 adolescents and 163 young adults completed the Self-Other Switching Task, a new computerised personality trait attribution paradigm. Measures of sustained attention, working memory, and inhibition were used to assess executive functioning. Linear mixed models showed that participants were faster at making attributions from the self-perspective and when referring to the self. They were also slower to disengage/switch from the self-perspective and the self-representation. Whereas there were no age differences in self-other switching efficiency per se, adolescents were slower than adults on trials involving appraisals of the other from the self-perspective. Importantly, higher verbal working memory scores were associated with better performance on incongruent trials and with switching scores. This study demonstrates the utility of a new experimental task permitting to tease apart the effects of self-other appraisal and perspective switching within a single paradigm. Our behavioural results highlight a self-cost observed in switching between representations and perspectives and emphasise the roles of age and working memory in the simultaneous processing of self- and other-oriented information.

## Introduction

Mentalizing is the imaginative capacity to understand one’s own and others’ behaviour in terms of underlying mental states, which is considered critical for communication and for navigating relationships (Allen et al., 2008; Apperly, 2021). This complex ability relies on a range of psychological processes, including self/other appraisal and perspective taking. We use the term self/other appraisal to refer to the general ability to activate mental representations of self and others, such as when evaluating personality traits (e.g., “*I am clumsy”* vs. “*My friend is funny*” illustrate self-other representations). Appraisal therefore would specify whether the target of mentalizing is the self or the other. We refer to perspective taking as the ability to make attributions of mental states from a specific point of view (e.g., “*I think my friend is funny*” vs. “My friend thinks he is funny” illustrate self vs. other perspectives). Thus, perspective would specify whether the subject/agent of mentalizing is the self or the other. In everyday mentalizing situations, these two processes allow us to switch between reflecting upon our own representations of others (“seeing others from the inside”) and others’ representations of ourselves (“seeing oneself from the outside”). In this study, we seek to better understand how appraising self and others, and doing so from the self and from the other’s perspectives operate together. We further aim to elucidate which factors can affect individual differences in self- and other-oriented mentalizing efficiency in adolescents and young adults.

The ability to mentalise self and others undergoes major development from adolescence to young adulthood. Among the hallmarks of adolescence is the development of the self-concept (Pfeifer & Peake, 2012; Sebastian et al., 2008), as well as the development of perspective taking abilities (Blakemore & Mills, 2014; Van der Graaff et al., 2014). Compared with childhood, self-representations in adolescence become more abstract and complex (Harter, 2015; Labouvie-Vief et al., 1995), and in parallel, there is increased consideration of others’ points of view, beliefs, and emotions (Hall et al., 2021). Importantly, the development of perspective taking also contributes to the development of a more differentiated self-concept, as it helps the adolescent integrate other’s beliefs about the self and contrast them to one’s own (Pfeifer et al., 2013; Pfeifer & Peake, 2012). Nonetheless, the differentiation between self and others can also be labile in adolescence, as peer judgements become increasingly important for establishing one’s identity (Pfeifer et al., 2009; Sebastian et al., 2008). Therefore, adolescence represents a particularly interesting developmental period to study individual differences in self-other mentalizing.

There are some arguments suggesting that appraisal and perspective taking, both with respect to self and others, can be seen as two distinct psychological processes, assessed by means of different behavioural paradigms and linked to distinct neural mechanisms. First, self/other appraisal, also referred to as self/other referential processing (Northoff et al., 2006), has been generally defined in the literature as the subjective estimation of one’s qualities or abilities, such as personality traits and is considered to be an essential part of self-concept development in adolescence (Crone et al., 2022). It is typically assessed using trait attribution paradigms, where participants are asked to evaluate themselves and others in terms of their personality traits or other characteristics (Heatherton et al., 2006; Kelley et al., 2002). Meta-analyses underline the role of the ventral medial prefrontal cortex (vmPFC) as the key brain region associated with representations of self and close others (Murray et al., 2012; van der Meer et al., 2010). On the contrary, perspective taking can be broadly defined as accessing somebody else’s point of view, which implies acknowledging that others have mental states (e.g., points of view, beliefs, emotions) that are different from our own; Erle & Topolinski, 2017). Its experimental study can involve not only visuo-spatial perspective taking paradigms (Keysar et al., 2003; Samson et al., 2010) but also theory of mind/belief attribution paradigms (Apperly, 2012; Baron-Cohen, 2001; Happé, 1994). Reflecting on other’s mental states typically yields activations within the “mentalizing brain network,” including the dorsolateral prefrontal cortex (dlPFC) and the temporoparietal junction (TPJ) (Bukowski, 2018; Schurz et al., 2013).

Albeit tapping into probably distinct psychological mechanisms and being assessed with distinct experimental methods, trait attribution and perspective taking paradigms also share a similarity as both reveal consistent behavioural indices differentiating self- versus other-oriented information processing. For instance, one of the most replicated findings in the literature is the self-advantage effect: the automaticity in the cognitive processing of self-representations (Maki & McCaul, 1985; Symons & Johnson, 1997) and self-perspectives (Back & Apperly, 2010; Bradford et al., 2015), characterised by faster and more accurate responses. On the contrary, mentalizing others is generally slower, more error-prone, and considered to demand more cognitive resources (Apperly et al., 2009; Birch & Bloom, 2007). Concordantly, several studies show that increasing executive load on mentalizing tasks can impede others’ mental state inference (Cane et al., 2017; Lin et al., 2010). Another important finding is that discrepancies in self- and other-oriented information can cause cognitive interference, requiring even more cognitive effort to resolve it. Incidentally, one’s own knowledge can bias the interpretation of others’ mental states. This effect is typically coined “egocentric interference” or “egocentric bias” (Apperly et al., 2010; Epley et al., 2004). Alternatively, others’ knowledge can also affect self-oriented inferences, thus producing “altercentric interference,” which is particularly likely if the other’s perspective is considered more relevant to the task (Samuel et al., 2020). Interestingly, some preliminary evidence reports increased egocentric and altercentric inference in adolescents (Weidema et al., 2023), suggesting that resolving self-other interference may be particularly challenging in adolescence.

The current literature leaves some important outstanding questions that we aim to address in the current study. First, as briefly reviewed above, self/other appraisal and perspective taking are typically studied separately, whereas, in real-life mentalizing, they operate together and interact. Indeed, everyday mentalizing involves *simultaneou*s, and not isolated processing of representations and perspectives. This is particularly important in adolescence, as perspective taking and self-concept development are deeply intertwined. Indeed, with the increased salience of peers and social comparison, adolescents start to integrate others’ representations about themselves to form their own (Crone et al., 2022; Rapee et al., 2019).

A few experimental studies have attempted to integrate activated representations and perspectives in trait attribution paradigms. For instance, research on self-concept development distinguishes direct self-appraisals, which refer to evaluations based on one’s own perspective (e.g., I think I am funny) and reflected self-appraisals, which refer to evaluations based on another person’s perspective (e.g., My friend thinks I am funny) (Pfeifer et al., 2007, 2009). In that way, direct and reflected appraisals allow one to contrast one’s own beliefs about the self and perceived beliefs of others about oneself. Studies in adolescents suggest that, compared with adults, adolescent self-representations may rely more heavily on others’ perspectives about the self (Pfeifer et al., 2009). Moreover, from early to late adolescence, direct and reflected self-appraisals become more aligned, which is evidenced by increased similarity in behavioural ratings as well as in associated mPFC activity (Pfeifer et al., 2013; van Buuren et al., 2022; Van der Cruijsen et al., 2019). Yet, existing research seems to focus specifically on appraisals of the self from different perspectives, which implies an experimental manipulation of the perspective, but not of the representation activated.

To the best of our knowledge, only one neuroimaging study to date (D’Argembeau et al., 2007) used a trait attribution paradigm that manipulated both the representation (self vs. close friend) and the perspective (self vs. close friend) activated during personality evaluations. In the task used, 17 young adults (mean age = 23 years) had to make four types of judgements on a series of personality adjectives. Two types of judgements involved making self- and other-evaluations from the first-person perspective (e.g., “Are you sociable?” “Is your friend sociable?”). The other two types of judgement involved making evaluations from the third person perspective (e.g., “According to your friend, are you sociable?” “According to your friend, is she sociable?”). The authors’ main goal was to disentangle the neural correlates of self-referential processing and perspective taking, and they indeed found that these two processes seem to activate different regions of the medial prefrontal cortex. The behavioural data showed a main effect of perspective: consistent with the self-advantage effect, subjects responded faster from their own perspective, than from their friend’s perspective. There was no effect of representation and no interaction between representation and perspective. The small sample of this neuroimaging study, however, hinders the interpretation of behavioural results and it remains to be clarified how different representation-perspective combinations affect reaction time in such paradigms.

Furthermore, everyday mentalizing also implies the ability to flexibly *switch* between self-other representations and perspectives. It remains yet unclear whether this switching process is relatively automatic or cognitively effortful. For example, a handful of studies specifically examined the cognitive cost of switching between the self-other perspectives, adopting visual perspective taking tasks (Ferguson et al., 2017), and belief attribution paradigms (Bradford et al., 2015, 2018, 2019). Together they showed that participants were generally faster to respond on trials where no perspective shift was required, whereas perspective shifting was associated with increased reaction time, synonymous to being cognitively effortful. Importantly, Bradford and colleagues (2015) also explored the effects of the direction of the perspective shift (from self to other vs. from other to self). They demonstrated that on consecutive trials involving a perspective shift from self to other, participants’ responses were slower and more error-prone when compared with shifting from other to self, supporting the automaticity of the self-perspective. Crucially, when there was no perspective switch across trials (self to self; other to other), there was no difference in performance between these trial types. It remains to be investigated whether similar switching costs can also be observed in trait attribution tasks when switching between self- and other-representations. In fact, more studies are needed to better characterise the process of self-other mentalizing costs across different experimental paradigms.

One additional consideration regarding self-other mentalizing is the extent to which domain-generic executive functions contribute to various aspects of self-other control. Dealing with self-other interference is likely to demand executive resources and there are in fact multiple reports of associations between perspective taking and executive functions (Carlson et al., 2002; Wardlow, 2013). Moreover, neuroimaging reviews underline how interference between self-other perspectives across different tasks activates brain regions involved in domain-general conflict resolution and attention, such as the posterior middle frontal gyrus, the inferior frontal gyrus, and the posterior dlPFC (Bukowski, 2018). However, the functional contributions of different facets of executive functioning with regard to self-other mentalizing remain to be clarified. For example, inhibitory control was previously associated with one’s susceptibility to interference effects from irrelevant perspectives (Ahmed & Miller, 2011; Lin et al., 2010; Qureshi et al., 2010), whereas working memory was linked specifically to the capacity of calculating and holding a given perspective in mind (Lin et al., 2010; Qureshi & Monk, 2018). Research also questions whether general attentional processes can explain performance on some mentalizing tasks (Heyes, 2014; Santiesteban et al., 2014) and this may be especially relevant for computer-based paradigms that require paying sustained attention to stimuli (Poznyak et al., 2019, 2024).

In the current study, we aim to address the outstanding questions raised above. Taking inspiration from a previously used personality trait attribution task (D’Argembeau et al., 2007; Schneider et al., 2012), we propose a novel experimental paradigm: the Self-Other Switching Task (SOST). Participants are asked to judge whether a given adjective accurately describes themselves or their best friend, from their own or from their friend’s perspective, while their response time is recorded. In that way, the task allows manipulation of both the representation and the perspective activated in each trial while assessing reaction time costs within trials (effects of representation-perspective combination: e.g., self-other vs. self-self) and between trials (switching effects: e.g., from self to other vs. from other to self).

Our primary objective is to test this new experimental task in a group of adolescents and young adults, exploring reaction time costs associated with the simultaneous switching of representations and perspectives. We expect to replicate the self-advantage effect and to find that switching trials are more costly than non-switching trials. Our secondary objective is to characterise how age and executive functions may contribute to the efficiency of self/other switching. We hypothesise that adolescents will be generally slower on trials demanding to judge what do they think about others versus themselves. Moreover, we expect adolescents to present a higher switch cost in comparison to adults. Finally, we aim to explore associations between SOST scores and measures of sustained attention, inhibition, and working memory, expecting to find positive associations between self-other flexibility indexes and executive functions. Given that executive functions undergo significant developments in adolescence (Crone & Steinbeis, 2017), we are also interested in exploring whether those associations may differ between adolescents and adults.

## Method

### Participants

Using a convenience sampling strategy, typically developing adolescents and young adults (*n* = 251) were recruited from the general population through advertisements of the research project. The recruitment was done by undergraduate psychology students at the University of Geneva as part of their course requirements, as well as by our research team members. Inclusion criteria were age (12–35 years), French language proficiency, and absence of current neurological or psychiatric diagnoses. Written informed consent was obtained from all participants and their legal guardians in the case of adolescent participants, in accordance with the ethics committee of the University of Geneva. The sample was divided into two groups depending on whether the participants were below or above 18 years of age. All participants identified as either male or female. The adolescent group comprised 88 subjects (59 female, age range 12–18; mean age = 15.9, *SD* = 1.28) and the adult group comprised 163 subjects (82 female, age range 22–31, mean age = 26.3, *SD* = 1.68).

### Measures

#### The SOST

This personality trait attribution paradigm was designed to evaluate the reaction time costs associated with switching between self/other representations and perspectives. The SOST was designed and presented on a PC using E-Prime 2.0.

**Figure 1.**
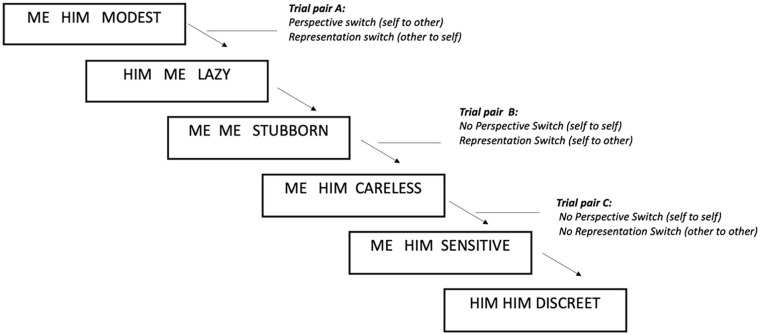
Example of the SOS experimental procedure. Each box represents one experimental trial. Trial pairs illustrate different types of switching in perspective and representation between two consecutive trials: Trial pair A = double-switch perspective and representation; Trial pair B = single-switch representation; Trial C = no switch in perspective or representation.

During task administration, participants are first instructed that they will be required to think about their best friend and always think about the same person for the whole duration of the task. They are then told that the task will consist in evaluating if (agree or disagree), from the perspective of either self or their friend, certain adjectives correspond to themselves or their friend. This evaluation will be initiated on the basis of three-word sentences presented on the screen, comprising two personal pronouns and an adjective (see illustration in Figure 1). The pronouns refer either to the participant (“I”) or to their best friend (“Him/Her,” depending on the friend’s gender). The first pronoun always represents the *perspective*, that is the person who attributes the adjective (self or other: “I think . . .”; “He/She thinks . . .”), and the second pronoun always represents the activated *representation*, that is the person to whom the adjective is attributed to aka the target of the judgement (self or other: “. . . I am modest”; “. . . He/She is stubborn”). The adjectives were selected from an existing research database of personality adjectives (Le Barbenchon et al., 2005): the aim was to include words that can be easily understood by young adolescents, based on the authors’ opinion. There was no objective evaluation of the valence of the adjectives. However, a consensus between the authors was reached to choose adjectives that had a generic positive or negative connotation. The chosen stimuli included three adjectives labelled by the authors as “positive” (discreet, modest, sensitive) and three adjectives labelled by the authors as “negative” (careless, lazy, stubborn). Participants are instructed to respond using the keyboard, if they agree (press N key) or disagree (press X key) with the statement displayed on the screen, as fast as possible. Thus, there are no correct or incorrect responses, as the judgement is subjective to the participant. Before starting the test phase, participants completed a training session of 16 trials and had the opportunity to ask the experimenter any questions.

The task follows a 2 × 2 × 2 repeated measures design with three conditions manipulated in each trial: Perspective (Self or Other), Representation (Self or Other), and Valence (Positive or Negative). This results in four possible within-trial perspective-representation conditions: two congruent (Self-perspective–Self-representation; Other perspective–Other representation) and two incongruent (Self-perspective–Other representation; Other perspective–Self representation). The task includes 130 trials in total: 32 stimuli presenting Self-Self statements, 32 stimuli presenting Self-Other statements, 33 stimuli presenting Other-Other statements, and 33 stimuli presenting Other-Self statements. Trials are separated by a short interval, marked by a fixation cross with a duration of 500 ms. The next trial appears immediately after the participant has pressed one of the response keys. The trials have been pseudo-randomised within four blocks. A series of consecutive trials can include “repeated” trials and “switching” trials. Repeated trials are defined as two consecutive trials where no change of perspective and/or representation has occurred. Switching trials, on the contrary, refer to a change of perspective and /or representation from one trial to another. Response time (in milliseconds) is recorded for each trial and represents the main dependent variable.

#### Executive function measures

##### Go-no-go task

The number-based version of this computerised sustained attention to response task (SART) was used (Robertson et al., 1997). Participants are presented with numbers from 1 to 9, one at a time, in a sequential manner. Participants are instructed to respond by pressing the Space bar every time a number is presented (go trials) on the screen, except when the number 3 appears (no-go trials). The task comprised three blocks. The first block represented a training session of 18 trials, followed by a break where participants could ask questions. The following two blocks comprised 226 trials in total, each trial separated by a 1,150 ms interval. Each number was presented for a duration of 250 ms, followed by a mask of 900 ms. The percentage of inhibition errors (response rate on the no-go trials) and omission errors (non-response rate on go trials) were used as variables of interest, assessing inhibition of automatic response and sustained attention, respectively.

##### Letter-number sequencing (LNS), subtest from the Wechsler Intelligence Scale for Children and Adults (WISC-V, WAIS-IV)

This subtest was used to assess working memory capacity. The experimenter reads out loud a series of letters (from A to Z) and numbers (from 0 to 9) and subjects are instructed to immediately recall this series saying the numbers first, in ascending order, and then the letters, in alphabetical order. The difficulty of the sequences increased progressively, with each subsequent item containing a larger combination of numbers and letters. A maximum of two points were awarded for each correctly ordered sequence, according to the scoring manual. The total accuracy score was then transformed into a scaled score based on normative data. The WISC-V version was used for adolescents (<18 years old) and the WAIS-IV was used for adults (≥18 years old). The scaled score obtained on this subtest was used for the statistical analysis.

### Procedure

The experimental procedure consisted of a single testing session lasting between 60 and 90 min. Participants completed the SOST task, the executive functioning measures outlined above, and a series of additional tests and questionnaires which are part of a larger experimental protocol and are not presented here. All measures were administered in a pre-defined order by postgraduate-level psychology students and/or research assistants.

### Data analysis

Data were first screened for outliers, defined as reaction times above or below 2.5 standard deviations from the grand mean, to account for anticipatory or very slow responses. A total of 833 of 32,630 data points were removed as outliers.

Data analysis consisted of four parts, focusing on **(a)** within-trial experimental conditions effects (perspective, representation, valence), **(b)** between-trials switching effects, **(c)** age moderations of within-trial conditions and between-trial switching effects, and **(d)** associations between individual differences in SOST response times and executive functioning scores, in the whole sample and in each age group. Analyses (a)–(c) were conducted within a single multilevel linear regression model, fitted to the trial-level data of the task. Analysis (d) was conducted using Pearson correlations on EF scores and individual performance measures derived from the multilevel model. The multilevel model was chosen due to its power to handle trial-level data, which was essential for modelling switching effects from one trial to the next. As well, the model allowed incorporation of continuous trial-varying covariates (e.g., trial number), to handle missing trials or conditions, and to model individual differences in task performance with so-called “random effects” (Fitzmaurice et al., 2012). Such options are not available for more traditional methods such as repeated measures analysis of variance (ANOVA).

#### Multilevel modelling

After screening and data cleaning, the trial-level data were entered into a multilevel linear regression with reaction time as the dependent variable. This model was fitted in two steps, with a strategy reducing model complexity only to necessary effects (i.e., Occam’s Razor principle).

In the first step, a full factorial design was included, accounting for perspective, representation, and valence effects for the current trial (i.e., experimental condition effects), in interaction with the same effects for the previous trial (i.e., switching effects). Of note, although valence effects are not of main interest for the current study, they were still controlled for in the model. In addition, to account for task habituation effects, the effect for the trial number was added, which represented adaptation in RT over the whole session (e.g., slowing down or speeding up as the task progresses). All variables were included as both fixed and random effects. Consequently, the model analysis further allowed us to account for individual differences with respect to all examined effects.

Once this initial model was fitted, a Type II ANOVA breakdown using *F*-tests was obtained to reduce the fixed and random effects to the most complex fixed interaction above the perspective × representation × valence three-way interaction. Of note, the task was not designed to test valence effects, and the degree of positivity and negativity of the adjectives was not controlled. However, as the task design included both positive and negative adjectives, we chose to include the possible effect of valence in the model. We opted for this strategy as a tradeoff between keeping the random effects design maximal, as recommended by literature (Barr, 2013), and reducing the computational burden imposed by complex random effects structures.

In the second step, the reduced design model was refitted while adding fixed moderation effects of age. Non-significant moderations were then removed to further reduce the model to its final form. For this model, fixed effects were inspected with a Type II breakdown using *F*-tests, with standardised regression coefficients as a measure of effect size. The final model was checked for its stability and statistical assumptions by inspecting collinearity diagnostics, influence diagnostics, residual diagnostics (linearity, normality, and homoscedasticity), and goodness-of-fit (see Supplementary Material for the list of diagnostic strategies and tests). Goodness-of-fit was quantified as marginal *R*^2^ and conditional *R*^2^, the former representing proportion-of-variance-explained (PVE) by the fixed effects (collapsing across random effects), and the latter representing PVE of fixed and random effects.

#### Correlational analysis

For the correlational analysis, we first derived individual differences in task performance from the final fitted multilevel model. These individual differences were obtained directly from the random effects, and represented for each subject (a) their grand average RT over all conditions, (b) their RT adaptation over trials, (c) their average RT for the four experimental conditions, (d) their within-trial perspective and representation contrasts (i.e., self vs. other), (e) their within-trial perspective-representation congruency contrast (i.e., self-self vs. self-other), (f) their average RT for between-trial perspective and representation switching conditions (i.e., self > other, other > self), and (g) their between-trial switching interference and congruency contrasts (i.e., self-other > self-self vs. self-other > other-self). Task individual differences were then correlated with the scores of executive function measures (Go-No-Go and WAIS-IV Letter-Number Sequencing), using Pearson correlations.

#### Technical specifications and software

All inferential tests were conducted at a reduced significance level of α = 0.005, following recent recommendations to reduce the number of false positive results in science (Benjamin et al., 2018). The estimation of multilevel models was done with maximum likelihood (ML) estimation. Degrees of freedom for *F*- and *t*-tests in the multilevel model were adjusted for random effects using the correction of Satterthwaite (Fitzmaurice et al., 2012). Continuous variables in the multilevel model were standardised to have a mean of 0 and a standard deviation of 1, to ensure the stability of the estimation algorithm.

All analyses were conducted using the R statistical software version 4.1.2 (R Core Team, 2020), with packages “lme4” for fitting multilevel regressions (Bates et al., 2014), “lmerTest” inferential tests in multilevel models (Kuznetsova et al., 2017), “car” for model diagnostics (Fox & Weisberg, 2019), “MuMIn” for calculation of marginal-*R*^2^ goodness-of-fit (MuMIn, 2020) “effectsize” for standardised regression parameters (Ben-Shachar et al., 2020), “visreg” for visualisation of multilevel effects (Breheny & Burchett, 2017), and “corrplot” for visualisation of correlations (Wei & Simko, 2017).

## Results

### Sample characteristics

Males and females did not differ in terms of age in the adolescent group, *t*(86) = –0.40, *p* = .6905, nor in the adult group, *t*(161) = 0.40, *p* = .6905. Age group differences in performance on executive measures are presented in Table 1. Adolescents showed significantly lower performance in comparison to adults on inhibition, sustained attention, and working memory scores.

**Table 1.** Means and standard deviations (*SD*) for go-no-go errors and WISC/WAIS letter-number sequencing, stratified by age group.

	Total (*N* = 251)	Adolescents (*N* = 88)	Adults (*N* = 163)	*t*-value (DF = 249)	*p*-value
Go-no-go % inhibition errors	47.53 (23.12)	52.93 (22.06)	44.61 (23.22)	2.75	0.0063
Go-no-go % omission errors	2.46 (4.18)	3.82 (5.53)	1.73 (3.01)	3.98	0.0001
WISC/WAIS-IV Letter-number sequencing scaled score	9.56 (2.89)	8.84 (3.16)	9.96 (2.67)	-2.94	0.0036

### Multilevel model

The final multilevel model that was fitted to the trial-level data contained 31 fixed effects in total, with one four-way interaction as its most complex effect. Model diagnostics revealed no problems with multicollinear parameters or heteroscedastic transformed residuals. Transformed residuals showed evidence of slight skewness towards large reaction times (i.e., positive skew), although this was not expected to affect inferential tests, due to the large sample size for the trial-level analysis. Goodness-of-fit for the model was calculated as a marginal *R*^2^ of 8.59%, and a conditional *R*^2^ of 45.8%, which qualified as “weak” to “moderate” explanatory power, respectively (Cohen, 2013). The large conditional *R*^2^ indicated individual differences in task performance (represented by random effects) accounted for a larger proportion of explained variance than population effects (represented by fixed effects). The fixed effects consisted of a number of different within-trial effects (i.e., experimental condition), between-trial effects (i.e., switch vs. no switch), and age moderations (see Table S3 in Supplementary Material for the full list).

#### Within-trial effects of perspective and representation

##### Main effects

A Type II ANOVA breakdown of the multilevel model revealed a significant main effect of perspective, such that mean RT was faster for self-perspective statements (*M* = 2,131.5 ms, *SE* = 45.0) than for other-perspective statements (*M* = 2,287.2 ms, *SE* = 49.6), *t*(288.2) = 10.81, *p* < .0001, **β*_z_* = 0.122. Similarly, there was a significant main effect of representation, such that mean RT was faster for self-representation statements (*M* = 2,138.7 ms, *SE* = 46.1) than for other-representation statements (*M* = 2,280.0 ms, *SE* = 48.7), *t*(275.7) = 9.32, *p* < .0001, *β_z_* = 0.111.

The main effect for valence was not significant, showing no significant difference in mean RT between positive and negative statements, *t*(1,257.0) = –0.03, *p* = .6180, *β_z_* = –0.005, and is not discussed further in this section. Finally, there was a significant main effect of trial, such that mean RT decreased by 7.5 ms for each increase by one trial (975 ms over 130 trials), *t*(250.5) = –22.257, *p* < .0001, *β_z_* = –0.2219, suggesting a habituation effect to the task.

##### Interactions

The ANOVA revealed a significant two-way interaction of perspective × representation, such that mean RT was faster for statements where perspective and representation were *congruent* (“self-self,” “other-other”; *M* = 2,018.71 ms, *SE* = 40.0), than for statements that were *incongruent* (“self-other,” “other-self”; *M* = 2,400.02 ms, *SE* = 50.8), *F*(1,252.5) = 372.83, *p* < .0001. No further interaction effects were found to be significant.

#### Between-trial effects of switching perspective and representation

##### Main effects

There were no significant main effects for the three variables characterising the previous trial (perspective_prev_, representation_prev_, valence_prev_), indicating that when taken in isolation, the previous trial conditions did not generally affect RT on the current trial.

##### Interactions

There was a significant two-way interaction of perspective_prev_ × perspective, *F*(1,296.9) = 72.93, *p* < .0001. Switching perspective was associated with higher reaction times compared with non-switching trials, *M_diff_* = 114.1 ms, *t*(250.4) = 8.84, *p* < .0001, *β_z_* = 0.09. Similarly, there was a significant interaction of representation_prev_ × representation, *F*(1,326.9) = 55.74, *p* < .0001. Switching representation was also associated with higher reaction times compared with repeated trials, *M_diff_* = 91.8 ms, *t*(252.7) = 7.83, *p* < .0001, *β_z_* = 0.07. There was no significant difference in RT associated with switching perspective or representation. Both were equally hard, compared with non-switching consecutive trials.

Importantly, the “Other to Self” switches were always faster than “Self to Other” switches, irrespective of whether the switch occurred in the perspective, *M_diff_* = –228.3 ms, *t*(251.6) = –7.84, *p* < .0001, *β_z_* = –0.18, or the representation, *M_diff_* = –183.6 ms, *t*(260.4) = –7.80, *p* < .0001, *β_z_* = –0.14.

For no-switch trials, responses were faster for repeated self-perspective trials, *M_diff_* = 275.4 ms, *t*(295.7) = 13.50, *p* < .0001, *β_z_* = 0.217, and repeated self-representation trials, *M_diff_* = 227.7 ms, *t*(303.7) = 11.49, *p* < .0001, *β_z_* = 0.179, compared with repeated other-perspective sequences, *M_diff_* = 36.1 ms, *t*(293.2) = 1.79, *p* = .0742, *β_z_* = 0.028, and repeated other-representation trials, *M_diff_* = 54.7 ms, *t*(284.6) = 2.73, *p* = .0067, *β_z_* = 0.043.

Finally, the ANOVA revealed a significant four-way interaction of perspective_prev_ × representation_prev_ × perspective × representation, *F*(1,340.6) = 62.56, *p* < .0001. On average, repeated trials were the fastest (*M* = 2,027.8, *SE* = 44.3), followed by single-switch (*M* = 2,227.5, *SE* = 45.8) and double-switch trials (*M* = 2,233.1, *SE* = 46.0). Whereas on average single-switch trials were not significantly faster than double-switch trials, *M_diff_* = –5.5 ms, *t*(3416.7) = –0.39, *p* = .6937, *β_z_* < –0.01, the slowest trials were those that double-switched an incongruent perspective-representation combination (i.e., “other-self > self-other” and “self-other > other-self”). The “other-self > self-other” trials were associated with slightly longer reaction times compared with “self-other > other-self” trials. However, the difference between these two trial types was only marginally significant, *M_diff_* = 31.2 ms, *t*(572.4) = 0.94, *p* = .0245, *β_z_* = 0.02.

For the repeated trials, this sequence congruency effect was greater for the “self-self > self-self” condition than for the “other-other > other-other” condition, *M_diff_* = 321.4, *t*(405.6) = 8.78, *p* < .0001, *β_z_* = 0.253.

#### Age effects

##### Main effects

The breakdown of the multilevel model revealed no significant main effect of age on reaction time.

##### Interactions

**Figure 2.**
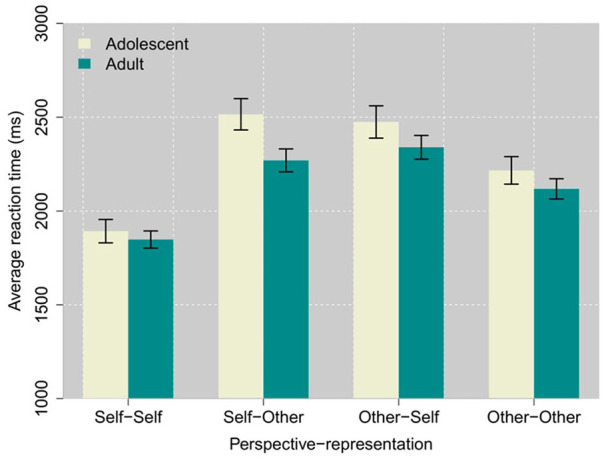
Within-trial performance on the SOST in adolescents and adults.

There was a significant two-way interaction of representation × age, *F*(1,303.5) = 8.17, *p* = .0046, such that overall, adolescents responded slower on trials with other representation than self-representation, *M_diff_* = 182.4 ms, *t*(301.8) = 7.63, *p* < .0001, *β_z_* = 0.143, compared with adults, *M_diff_* = 100.1 ms, *t*(289.7) = 5.72, *p* < .0001, *β_z_* = 0.079. Importantly, the ANOVA also revealed a significant three-way interaction of perspective × representation × age, *F*(1,255.4) = 9.54, *p* = .0022, such that interference for incongruent statements (“self-other,” “other-self”) was greater for adolescents (*M* = 2,495.2 ms, *SE* = 67.1) than for adults (*M* = 2,304.9 ms, *SE* = 49.4). Contrast comparisons showed that the above interactions appear to be driven specifically by slower reaction times observed on the “self-other” condition in adolescents (See Figure 2). Single- or double-switch RT costs were not modified by age.

As the sample size differed between adolescents and adults, we performed additional exploratory analyses to ensure the robustness of the findings. The analysis was repeated 10 times, each time randomly selecting a sub-group of adults to match the adolescent group in terms of size. Results indicated that nearly all the effects that were significant at α = 0.005 in the original model remained so in 10 out of 10 re-analyses.

### Correlations between self-other switching efficiency and executive functioning

Results revealed significant negative correlations between SOST grand average RT in the SOST and the working memory (LNS) measure, *r* = –.17, *t*(245) = –2.85, *p* = .0048, such that faster participants tended to have a higher working memory score. Similarly, faster responses on the incongruent perspective-representation trials (i.e., “other-self” and “self-other”) were also associated with better working memory scores, *r* = –.18, *t*(245) = –2.90, *p* = .0040, for “other-self” trials, and *r* = –.20, *t*(245) = –3.22, *p* = .0015, for “self-other” trials. In general, larger negative congruency contrasts were associated with lower LNS values, *r* = 0.18, *t*(245) = 3.00, *p* = .0029.

Furthermore, higher working memory scores were correlated with faster average RT for “self > other” representation switching, *r* = –.19, *t*(245) = –3.04, *p* = .0026, as well as for “other-self > self-other” perspective-representation switching, *r* = –.20, *t*(245) = –3.17, *p* = .0017. However, no correlation was observed between SOST performance and Go-No-Go indices of sustained attention and inhibition (all *p* > .005). The correlation analysis was repeated for adolescents and adults separately. This revealed no change in correlation structure between task performance and cognitive measures. For within-task performance measures, however, individual differences were more strongly intercorrelated for adults than adolescents (see Supplementary Material).

## Discussion

This study introduced a novel experimental paradigm—the SOST—designed to assess reaction time costs associated with simultaneous processing of and switching between two key psychological processes in mentalizing, self/other appraisals and perspective taking. We aimed to characterise individual differences in performance on the SOST, with a particular focus on developmental differences between adolescents and adults as well as potential associations with executive functioning. The individual variations in reaction times observed on the SOST can be summarised by three principal task effects: self-advantage, congruency, and switching. In this section, we consider each effect in turn, discussing the effects of age and the relationships detected between task scores and measures of executive functioning.

### Self-advantage effect

The first line of results demonstrated that typically developing adolescents and adults are faster to reflect *from* their own perspective as well as *about* themselves. On the contrary, activating others’ perspectives and/or representations appears to engage more cognitive effort, as evidenced by longer response times. This highlights the psychological distinction between automatic self-oriented processing and the more cognitively costly other-oriented processing both during appraisal and perspective taking. Our findings on the SOST are in agreement with the literature emphasising the automaticity of self-oriented information processing (Antshel et al., 2010; Back & Apperly, 2010; Bradford et al., 2015, 2019; Heatherton et al., 2006; Schneider et al., 2012) and are supported by neuroimaging studies that point to distinguishable, albeit partly overlapping neural correlates of self and other-related processing in adolescents and adults (Kelley et al., 2002; Murray et al., 2012, 2015; Northoff et al., 2006).

### Congruency effect

The second major line of results can be understood in terms of congruency effects: in the whole sample, reaction times on incongruent trials, where the perspective and representation differed, were slower than on congruent trials, where the perspective and representation were the same. Thus, simultaneous processing of incongruent perspectives and representations appears to be particularly costly and according to our correlational results, may rely on working memory capacities. We propose that working memory is likely to be implicated in simultaneously holding in mind self- and other-oriented information, especially as the SOST requires online processing of verbal material. This is in line with another report showing associations between a language-based perspective taking task and working memory (Ryskin et al., 2015).

Interestingly, response times on incongruent trials were particularly slower for adolescents, compared with adults. Evaluating others’ from their own perspective was especially effortful for younger participants, which can suggest a heightened uncertainty about and perhaps particular significance of self-other attributions in adolescents. This supports the idea that adolescence is a critical time for the development of higher-order socio-cognitive abilities and perspective taking, as perceptions of self and others change (Blakemore & Mills, 2014). Figuring out “*what I think about my friends*” and “*what my friends think about me*” entails comparing oneself to others and becomes an essential part of finding one’s place among peers. Importantly, such social comparisons further contribute to the development of the self-concept in adolescence (Crone & Fuligni, 2020). The amplified incongruency effect observed especially on the “Self-Other” condition in adolescents could thus reflect the personal relevance that induce trials involving social comparisons at that age. More generally, longer reaction times observed in adolescents could reflect the ongoing development of mentalizing and perspective taking abilities (Jankowski et al., 2014; Keulers et al., 2010; Klapwijk et al., 2013).

### Switching effects

As expected, we demonstrated that compared with repeated trials, switching trials were associated with longer reaction times, highlighting that switching between self-other polarities is cognitively effortful (Debbané et al., 2017). Interestingly, this was irrespective of whether the switch occurred in the perspective or the representation. Thus, both types of switches appear to demand similar cognitive effort.

Importantly, individual differences in switching costs were intertwined with the self-advantage and congruency effects discussed earlier. First, we showed that switching from Other to Self, in both perspective and representations switches, was faster than from Self to Other. With this, we replicate and extend Bradford et al. (2015) findings by showing that a reaction time cost is observed when disengaging from the self-perspective, but also from self-representations. This suggests that it is cognitively costly to switch from self-oriented information in general. Thus, the automaticity of self-processing does not only imply advantages but also results in cognitive costs when it comes to disengaging from one’s own point of view and from self-appraisals. Second, switches from one incongruent trial to another (Self-Other > Other-Self and Other-Self > Self-Other) were the costliest in all participants as in these trial combinations, the incongruency effect essentially doubles.

Furthermore, switching costs showed negative correlations with working memory in both adolescents and adults. Higher working memory scores were associated with faster switches from self to other representation and with the “Other-Self > Self-Other” perspective-representation switch. The self-cost in representation switching was thus reduced for participants with higher working memory scores. Interestingly, we did not find any associations between self-other switching costs and measures of sustained attention and inhibition. Perhaps the lack of associations could imply that these dimensions of EF were not predominantly recruited during the execution of our experimental task (Cook, 2014). For example, inhibitory control has been previously associated specifically with interference from different perspectives, when participants must inhibit an irrelevant point of view to infer a correct one (Qureshi et al., 2010). The SOST, however, did not require to produce a correct inference of a mental state, or resolve conflicting perspectives, which could explain the lack of associations with inhibition. Indeed, the major aim of the task was to assess the process and not the outcome of mentalizing. We hypothesise that the SOST was much more demanding in terms of working memory capacities, as the trait attribution paradigm required to hold in mind different perspectives and representations. In addition, it implied an online “translation” of the presented stimuli into a full sentence (“ME HIM LAZY” has to be interpreted as “I think George is lazy”), which required mental manipulation of verbal information. Overall, the implication of executive functions in self-other control and more generally in social cognition appears worthy of further scrutiny (Bradford et al., 2015; Carlson et al., 2004). Indeed, both are conceptualised as multidimensional higher-order functions and it is yet unclear which dimensions of EF are particularly associated with which components of social cognition. Further investigation of the relationship between self-other switching and executive functions, across different task demands, is needed.

Against our initial hypothesis, we did not observe developmental differences in self-other switching efficiency. In fact, switching efficiency in adolescents seemed to be driven mainly by the increased incongruency sensitivity on the “self-other” trials. When we control for this, adolescents do not appear to be slower switchers compared with adults. Therefore, it appears that it is primarily the changes occurring in the development of the self-other appraisals and perspective taking that distinguished switching efficiency on the SOST between adolescents and adults. Furthermore, it is important to note that the different SOST scores were more strongly intercorrelated in adults, suggesting that in adolescents there was more inter-individual variability affecting performance. Given that executive scores were also overall lower in adolescents, we can speculate that in adolescents, it may be the integration between the simultaneous development of different abilities such as perspective taking and executive functions that contributes to the flexibility involved in reasoning about self and others. In line with this, in their recent neurocognitive model of self-concept development, E. Crone and colleagues propose that cognitive advancements including perspective taking and social comparison differentially mediate self-concept development (Crone et al., 2022). Specific factors contributing to individual differences in self-other reasoning in adolescence as well as their relationships to major milestones in socio-emotional development remain to be further explored.

### Limitations and future directions

One important limitation of our study is the smaller sample of adolescents, compared with that of adults. Moreover, our study was cross-sectional, which did not enable us to draw any firm conclusions about the development of self-other control processes from adolescence to adulthood. Larger studies, including both cross-sectional and longitudinal designs, with age ranges from late childhood to adulthood are needed to specifically address differences in self-other control abilities across the lifespan. Longitudinal studies would be particularly beneficial to characterise the interactions between the developmental trajectories of executive functioning, self/other appraisal, and perspective taking. Another limitation is that, based on prior literature, we focused solely on associations with sustained attention, inhibition, and working memory. This experiment would have benefitted from including a measure of shifting (Miyake et al., 2000) to test the associations between the self-other switching in our task and domain-general mental flexibility. Indeed, it can be hypothesised that better cognitive flexibility abilities can also be associated with more efficient switching between self and other-oriented information (Cai & Qi, 2023).

Another important methodological limitation is that the SOST was not designed to reliably assess the effects of positive and negative valence during the trait attribution task. However, there is some indication of developmental differences in positive and negative self-evaluations that could be explored further (Van der Aar et al., 2018). Future studies should include more refined tasks that include adjectives of established positive and negative valence, as confirmed by subjective ratings of adolescents and adults from the general population.

Finally, future research could adapt the SOST to explore self-other distinction in clinical populations. Indeed, research in clinical psychology and psychiatry has demonstrated that a number of clinical conditions are associated with disruptions in self-other information processing; among others are borderline personality disorder (Ballespí et al., 2021; De Meulemeester et al., 2021) and autism spectrum disorders (Lamm et al., 2016). Another important avenue for future research would be to test the SOST using neuroimaging methods. Exploring neural activations during a task that manipulates both perspective taking and representation activation, as well as the directions of switching between them could provide further insights into neural correlates implicated in the self-other distinction across development (Murray et al., 2012, 2015).

## Conclusion

In summary, the present study demonstrated that the SOST can be reliably used to assess individual differences in self-other perspective and representation switching in adolescents and adults from the general population. Consistent with prior literature, results highlighted the self-advantage effect: participants were faster to make judgements from the self-perspective and about the self. Importantly, they were also slower to disengage/switch from the self-perspective and representations. Furthermore, whereas there were no age differences in self-other switching efficiency per se, adolescents were slower on trials involving incongruent perspective and representation and self-other statements in particular, which supports the ongoing development of the self-concept and perspective taking in adolescence. Finally, our results stress the role of working memory in simultaneously holding in mind both self- and other-oriented information, as well as in switching between perspectives and representations.

## Supplemental Material

sj-docx-1-qjp-10.1177_17470218241311415 – Supplemental material for Unpacking mentalizing: The roles of age and executive functioning in self-other appraisal and perspective taking Supplemental material, sj-docx-1-qjp-10.1177_17470218241311415 for Unpacking mentalizing: The roles of age and executive functioning in self-other appraisal and perspective taking by Elena Poznyak, Lucien Rochat, Deborah Badoud, Ben Meuleman and Martin Debbané in Quarterly Journal of Experimental Psychology

sj-pdf-1-qjp-10.1177_17470218241311415 – Supplemental material for Unpacking mentalizing: The roles of age and executive functioning in self-other appraisal and perspective taking Supplemental material, sj-pdf-1-qjp-10.1177_17470218241311415 for Unpacking mentalizing: The roles of age and executive functioning in self-other appraisal and perspective taking by Elena Poznyak, Lucien Rochat, Deborah Badoud, Ben Meuleman and Martin Debbané in Quarterly Journal of Experimental Psychology
