# Supplementary material for: Unpacking mentalizing: The roles of age and executive functioning in self-other appraisal and perspective taking
Source: Q J Exp Psychol (Hove). 2025 Jan 8;78(8):1707–20. doi: 10.1177/17470218241311415 (PMC12267865; doi:10.1177/17470218241311415)
Supplement: sj-docx-1-qjp-10.1177_17470218241311415 – Supplemental material for Unpacking mentalizing: The roles of age and executive functioning in self-other appraisal and perspective taking [file sj-docx-1-qjp-10.1177_17470218241311415.docx]

**Supplementary Material for:**

**Title: Unpacking mentalizing: the roles of age and executive functioning in self-other appraisal and perspective taking.**

Elena Poznyak^a*^, Lucien Rochat^b,c^, Deborah Badoud^a^, Ben Meuleman^d,e^, Martin Debbané^a,f^.

^a^ Developmental Clinical Psychology Research Unit, Faculty of Psychology and Educational Sciences, University of Geneva, Switzerland

^b^ Specialized Facility in Behavioral Addiction ReConnecte, Department of Mental Health and Psychiatry, University Hospitals of Geneva, Geneva, Switzerland

^c^ Faculty of Psychology, UniDistance, Brig, Switzerland

^d^ Swiss Center for Affective Sciences, University of Geneva, Geneva, Switzerland

^e^ Faculty of Psychology and Educational Sciences, University of Geneva, Switzerland

^f^ Research Department of Clinical, Educational and Health Psychology, University College London, United Kingdom

*Corresponding author: Elena Poznyak, Developmental Clinical Psychology Research Unit, Faculty of Psychology and Educational Sciences, University of Geneva, Switzerland

40 Boulevard du Pont d’Arve

1205 Geneva, Switzerland

e-mail address: [elena.poznyak@unige.ch](mailto:elena.poznyak@unige.ch)

Tel: +41223799345

**S1 Multilevel modelling technical specifications**

After screening and data cleaning, the trial-level data were entered into a multilevel linear regression with reaction time as the continuous dependent variable. This model was fitted in 2 steps.

In the first step, a full factorial task and switching design was included, both as fixed and random effects, consisting of perspective_prev_ × representation_prev_ × valence_prev_ × perspective × representation × valence. This design accounted for perspective, representation, and valence effects for the current trial (i.e., task effects), in interaction with the same effects for the previous trial (i.e., switching effects). In addition, a fixed and random effect was added for the trial number, and which represented adaptation in RT over the whole session (e.g., slowing down or speeding up). By having the same design both as fixed and as random effects, the model analysis further allowed that individual participants differed with respect to all of these task and switching effects (e.g. some participants are slower at switching). Once this initial model was fitted, a Type II ANOVA breakdown using *F*-tests was obtained to reduce the fixed and random effects to the most complex fixed interaction above the perspective × representation × valence three-way interaction. We opted for this strategy as a tradeoff between keeping the random effects design maximal, as recommended by literature (Barr et al., 2013), and reducing the computational burden imposed by complex random effects structures (e.g., taking days and weeks to estimate).

In the second step, the reduced design model was refitted while adding fixed moderation effects of age and gender.^[[1]](#footnote-1)^ Non-significant moderations were then removed to further reduce the model to its final form. In R, the final model formula used in the multilevel function of package lme4 as thus as follows:

rt_z ~ trial_z + (persp_prev + repr_prev + val_prev +

persp + repr + val + persp_prev:repr_prev +

persp_prev:persp + persp_prev:repr + persp_prev:val +

repr_prev:persp + repr_prev:repr + val_prev:repr +

val_prev:val + persp:repr + persp:val +

repr:val)*(age+gender) +

persp_prev:repr_prev:persp + persp_prev:repr_prev:repr +

persp_prev:persp:repr + persp_prev:persp:val +

repr_prev:persp:repr + val_prev:repr:val +

persp:repr:val + persp_prev:repr_prev:persp:repr +

(1+ persp_prev + repr_prev + val_prev +

persp + repr + val + trial_z + persp_prev:repr_prev +

persp_prev:persp + persp_prev:repr + persp_prev:val +

repr_prev:persp + repr_prev:repr + val_prev:repr +

val_prev:val + persp:repr + persp:val +

repr:val + persp_prev:repr_prev:persp + persp_prev:repr_prev:repr +

persp_prev:persp:repr + persp_prev:persp:val +

repr_prev:persp:repr + val_prev:repr:val +

persp:repr:val + persp_prev:repr_prev:persp:repr | id )

For this model, fixed effects were inspected with a Type II breakdown using *F*-tests, while random effects were inspected with a Type II breakdown using likelihood ratio tests. As a measure of effect size for fixed effects, we calculated standardized regression coefficients. The final model was also checked for its stability and statistical assumptions by inspecting (a) collinearity diagnostics, (b) influence diagnostics, (c) residual diagnostics (linearity, normality, and homoscedasticity), and (d) goodness-of-fit. For collinearity, we inspected variance inflation factors (VIF) for fixed effects, removing any IVs with a VIF that exceeded 10 (Kutner et al., 2005). For influence diagnostics, we inspected boxplots of model residuals and random effects, the former for identifying outlying cases, the latter for identifying outlying subjects. For residual diagnostics, we first computed decorrelated residuals according to the procedure recommended by Fitzmaurice, Laird, and Ware (2004). Then, we plotted these transformed residuals against transformed fitted values to check for issues with non-normality and non-constant variance (i.e., heteroscedasticity). Finally, goodness-of-fit was quantified using marginal-*R^2^*, which quantifies the proportion of variance in the outcome explained by the fixed effects, collapsing across random effects.

**S2 Supplemental model-based descriptive statistics**

**Table S1.** Means and standard errors (SE) for Perspective × Representation conditions

|  |  | **Mean (SE)** | |
| --- | --- | --- | --- |
| **Perspective** | **Representation** | **Adolescents** | **Adults** |
| *Self* | *Self* | 1892.6 (62.5) | 1847.8 (45.8) |
| *Self* | *Other* | 2515.7 (83.5) | 2269.9 (61.2) |
| *Other* | *Self* | 2474.7 (86.2) | 2339.8 (63.1) |
| *Other* | *Other* | 2216.5 (73.5) | 2117.9 (53.8) |

**Table S2.** Means and standard errors (SE) for Perspective and Representation switching conditions, stratified by Age.

|  |  |  | **Mean (SE)** | |
| --- | --- | --- | --- | --- |
| **Switch** | **Prev. perspective** | **Perspective** | **Adolescents** | **Adults** |
| *No* | *Self* | *Self* | 2136.5 (69.2) | 1997.0 (50.6) |
|  | *Other* | *Other* | 2279.8 (79.5) | 2184.9 (58.29 |
| *Yes* | *Self* | *Other* | 2411.4 (79.2) | 2272.9 (58.1) |
|  | *Other* | *Self* | 2271.8 (75.3) | 2120.7 (55.3) |
| **Switch** | **Prev. perspective** | **Perspective** | **Adolescents** | **Adults** |
| *No* | *Self* | *Self* | 2150.2 (74.9) | 2050.1 (54.8) |
|  | *Other* | *Other* | 2315.1 (77.2) | 2148.9 (56.7) |
| *Yes* | *Self* | *Other* | 2417.0 (78.5) | 2238.9 (56.7) |
|  | *Other* | *Self* | 2217.1 (72.8) | 2137.5 (53.3) |

**S3 Supplemental effect tables and graphs**

**Table S3.** Type II ANOVA breakdown of effects in the multilevel model.

| **Group** | **Effect** | **DF** | **F-value** | **P-value** |
| --- | --- | --- | --- | --- |
| Within-trial effects | trial | (1,250.5) | 495.37 | < 0.0001 |
|  | perspective | (1,284.1) | 135.66 | < 0.0001 |
|  | representation | (1,268.3) | 80.12 | < 0.0001 |
|  | valence | (1,254.0) | 0.25 | 0.6180 |
|  | perspective × representation | (1,252.5) | 372.83 | < 0.0001 |
|  | perspective × valence | (1,463.6) | 0.08 | 0.7758 |
|  | representation × valence | (1,273.3) | 6.54 | 0.0111 |
|  | perspective × representation × valence | (1,420.1) | 0.03 | 0.8675 |
|  |  |  |  |  |
| Between-trial effects | perspective_prev_ | (1,428.4) | 1.21 | 0.2723 |
|  | representation_prev_ | (1,437.8) | 0.40 | 0.5286 |
|  | valence_prev_ | (1,384.5) | 1.87 | 0.1723 |
|  | perspective_prev_ × representation_prev_ | (1,532.5) | 3.24 | 0.0725 |
|  | perspective_prev_ × perspective | (1,296.9) | 72.93 | < 0.0001 |
|  | perspective_prev_ × representation | (1,361.0) | 0.07 | 0.7969 |
|  | perspective_prev_ × valence | (1,365.6) | 1.86 | 0.1732 |
|  | representation_prev_ × perspective | (1,499.5) | 0.12 | 0.7264 |
|  | representation_prev_ × representation | (1,326.9) | 55.74 | < 0.0001 |
|  | valence_prev_ × representation | (1,438.4) | 0.73 | 0.3935 |
|  | valence_prev_ × valence | (1,353.3) | 0.03 | 0.8594 |
|  | perspective_prev_ × representation_prev_ × perspective | (1,419.4) | 0.38 | 0.5354 |
|  | perspective_prev_ × representation_prev_ × representation | (1,439.4) | 0.31 | 0.5791 |
|  | perspective_prev_ × perspective × representation | (1,368.5) | 0.20 | 0.6563 |
|  | perspective_prev_ × perspective × valence | (1,413.4) | 2.15 | 0.1435 |
|  | representation_prev_ × perspective × representation | (1,717.7) | 2.58 | 0.1084 |
|  | valence_prev_ × representation × valence | (1,326.5) | 8.60 | 0.0036 |
|  | perspective_prev_ × representation_prev_ × perspective × representation | (1,340.6) | 62.56 | < 0.0001 |
|  |  |  |  |  |
| Age moderations | age | (1,251.7) | 2.17 | 0.1417 |
|  | perspective × age | (1,301.4) | 1.01 | 0.3166 |
|  | representation × age | (1,303.5) | 8.17 | 0.0046 |
|  | perspective × representation × age | (1,255.4) | 9.54 | 0.0022 |
|  | perspective_prev_ × perspective × age | (1.340.8) | 0.99 | 0.3216 |
|  | representation_prev_ × representation × age | (1.358.7) | 0.03 | 0.8647 |
|  | perspective_prev_ × representation_prev_ × perspective × representation × age | (1.264.6) | 0.00 | 0.9570 |
|  |  |  |  |  |

**Figure S1. Reaction times observed when switching perspective and representation, respectively, between consecutive trials in adolescents and adults.**


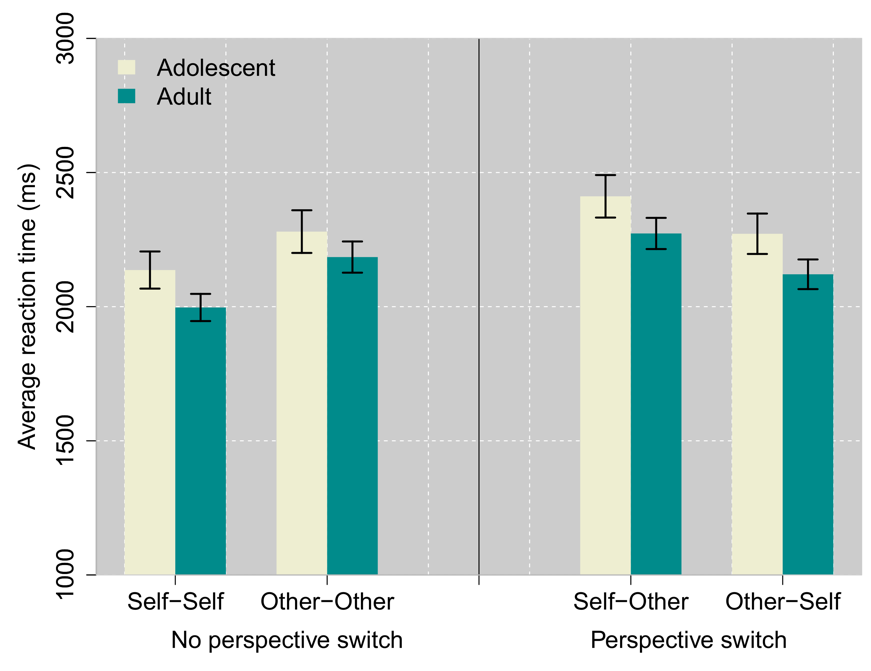


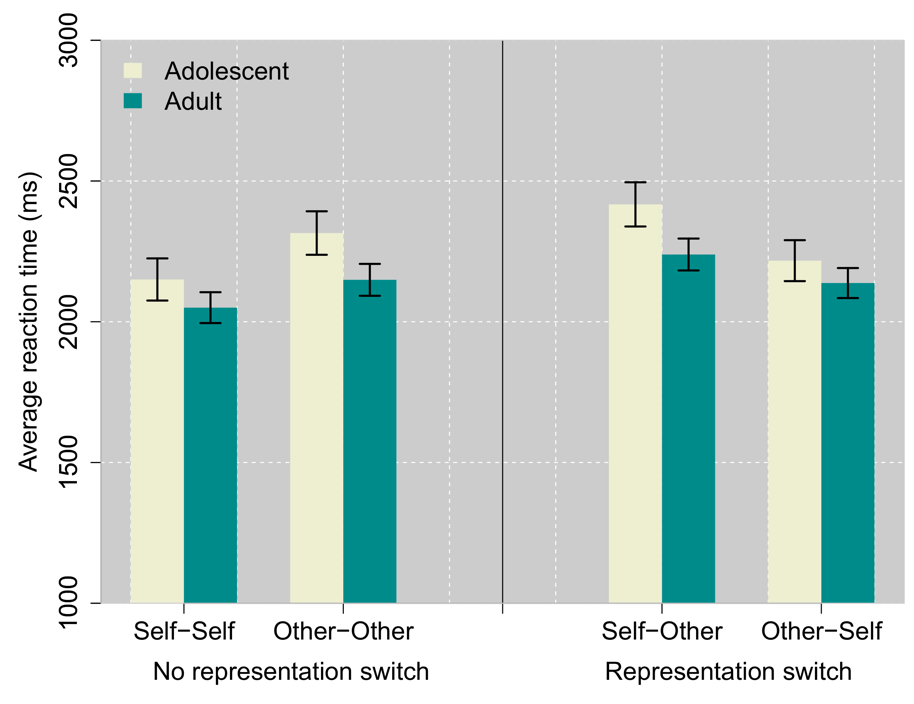


**References**

Barton, K. (2020). MuMIn: Multi-Model Inference. R package version 1.43.17.

Barr, D. J., Levy, R., Scheepers, C., & Tily, H. J. (2013). Random effects structure for confirmatory hypothesis testing: Keep it maximal. *Journal of memory and language*, 68(3), 255–278.

Fitzmaurice, G.M., Laird, N.M., and Ware, J.H. (2012). *Applied Longitudinal Analysis*. John Wiley & Sons.

Kutner, M. H., Nachtsheim, C. J., Neter, J., & Li, W. (2005). *Applied Linear Statistical Models.* McGraw-hill.

1. Age and gender cannot be random effects due to being between-subjects measures, not within-subjects measures. [↑](#footnote-ref-1)
