# Supplementary material for: Unpacking mentalizing: The roles of age and executive functioning in self-other appraisal and perspective taking
Source: Q J Exp Psychol (Hove). 2025 Jan 8;78(8):1707–20. doi: 10.1177/17470218241311415 (PMC12267865; doi:10.1177/17470218241311415)

Supplementary material: Results of the correlational analysis

Correlations whole sample

	RT_grand	RT_OO	RT_OS	RT_SO	RT_SS	RT_perspswitch_OS	RT_perspswitch_SO	RT_reprswitch_OS	RT_reprswitch_SO	RT_persp_egocentric	RT_repr_egocentric	RT_persprepr_congruency	RT_persp_switch_interference	RT_repr_switch_interference	RT_persp_switch_congr	RT_repr_switch_congr	RT_switch_OOSS	RT_switch_OSSO	RT_switch_SOOS	RT_switch_SSOO	RT_adaptation	RT_switch_congruency	GONOGO_error_inhib	GONOGO_ommission	GONOGO_SD	SeqLC_SD
RT_grand	×	96	98	97	95	97	98	98	98	33	19	−58	×	−23	×	×	85	93	90	88	×	−38	×	×	×	−18
RT_OO	96	×	91	90	89	92	96	91	97	47	34	−42	−27	−37	−19	×	82	86	83	93	×	−49	×	×	×	×
RT_OS	98	91	×	94	90	93	96	97	94	41	×	−67	−21	×	×	×	80	90	91	83	×	−35	×	×	×	−18
RT_SO	97	90	94	×	88	96	94	93	96	20	32	−70	×	−28	×	×	79	95	88	81	×	−38	×	×	×	−20
RT_SS	95	89	90	88	×	95	90	95	91	×	×	−39	×	×	×	×	91	86	83	84	×	−22	×	×	×	×
RT_perspswitch_OS	97	92	93	96	95	×	94	96	95	19	18	−55	×	−20	−29	×	90	94	88	84	×	−35	×	×	×	×
RT_perspswitch_SO	98	96	96	94	90	94	×	95	96	43	23	−55	−31	−26	−28	×	82	89	91	91	×	−46	×	×	×	×
RT_reprswitch_OS	98	91	97	93	95	96	95	×	94	29	×	−57	×	×	×	×	88	91	92	84	×	−33	×	×	×	×
RT_reprswitch_SO	98	97	94	96	91	95	96	94	×	35	30	−55	×	−38	−18	×	83	93	87	92	×	−45	×	×	×	−19
RT_persp_egocentric	33	47	41	20	×	19	43	29	35	×	×	×	−72	−25	×	×	×	18	32	45	×	−47	×	×	×	×
RT_repr_egocentric	19	34	×	32	×	18	23	×	30	×	×	×	−18	−73	−18	×	×	25	×	28	×	−52	×	×	×	×
RT_persprepr_congruency	−58	−42	−67	−70	−39	−55	−55	−57	−55	×	×	×	×	×	×	×	−31	−66	−63	−32	×	22	×	×	×	19
RT_persp_switch_interference	×	−27	−21	×	×	×	−31	×	×	−72	−18	×	×	19	×	×	×	×	−23	−33	×	36	×	×	×	×
RT_repr_switch_interference	−23	−37	×	−28	×	−20	−26	×	−38	−25	−73	×	19	×	×	×	×	−26	×	−43	×	43	×	×	×	×
RT_persp_switch_congr	×	−19	×	×	×	−29	−28	×	−18	×	−18	×	×	×	×	×	−32	−23	−27	−24	×	33	×	×	×	×
RT_repr_switch_congr	×	×	×	×	×	×	×	×	×	×	×	×	×	×	×	×	×	×	×	×	×	18	×	×	×	×
RT_switch_OOSS	85	82	80	79	91	90	82	88	83	×	×	−31	×	×	−32	×	×	79	76	77	×	−23	×	×	×	×
RT_switch_OSSO	93	86	90	95	86	94	89	91	93	18	25	−66	×	−26	−23	×	79	×	86	77	×	−31	×	×	×	−20
RT_switch_SOOS	90	83	91	88	83	88	91	92	87	32	×	−63	−23	×	−27	×	76	86	×	75	×	−24	×	×	×	×
RT_switch_SSOO	88	93	83	81	84	84	91	84	92	45	28	−32	−33	−43	−24	×	77	77	75	×	×	−47	×	×	×	×
RT_adaptation	×	×	×	×	×	×	×	×	×	×	×	×	×	×	×	×	×	×	×	×	×	×	×	×	×	×
RT_switch_congruency	−38	−49	−35	−38	−22	−35	−46	−33	−45	−47	−52	22	36	43	33	18	−23	−31	−24	−47	×	×	×	×	×	×
GONOGO_error_inhib	×	×	×	×	×	×	×	×	×	×	×	×	×	×	×	×	×	×	×	×	×	×	×	46	18	×
GONOGO_ommission	×	×	×	×	×	×	×	×	×	×	×	×	×	×	×	×	×	×	×	×	×	×	46	×	72	×
GONOGO_SD	×	×	×	×	×	×	×	×	×	×	×	×	×	×	×	×	×	×	×	×	×	×	18	72	×	−22
SeqLC_SD	−18	×	−18	−20	×	×	×	×	−19	×	×	19	×	×	×	×	×	−20	×	×	×	×	×	×	−22	×

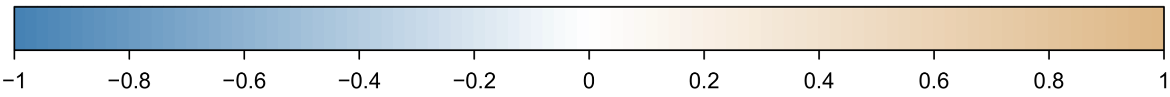

Correlations adolescents

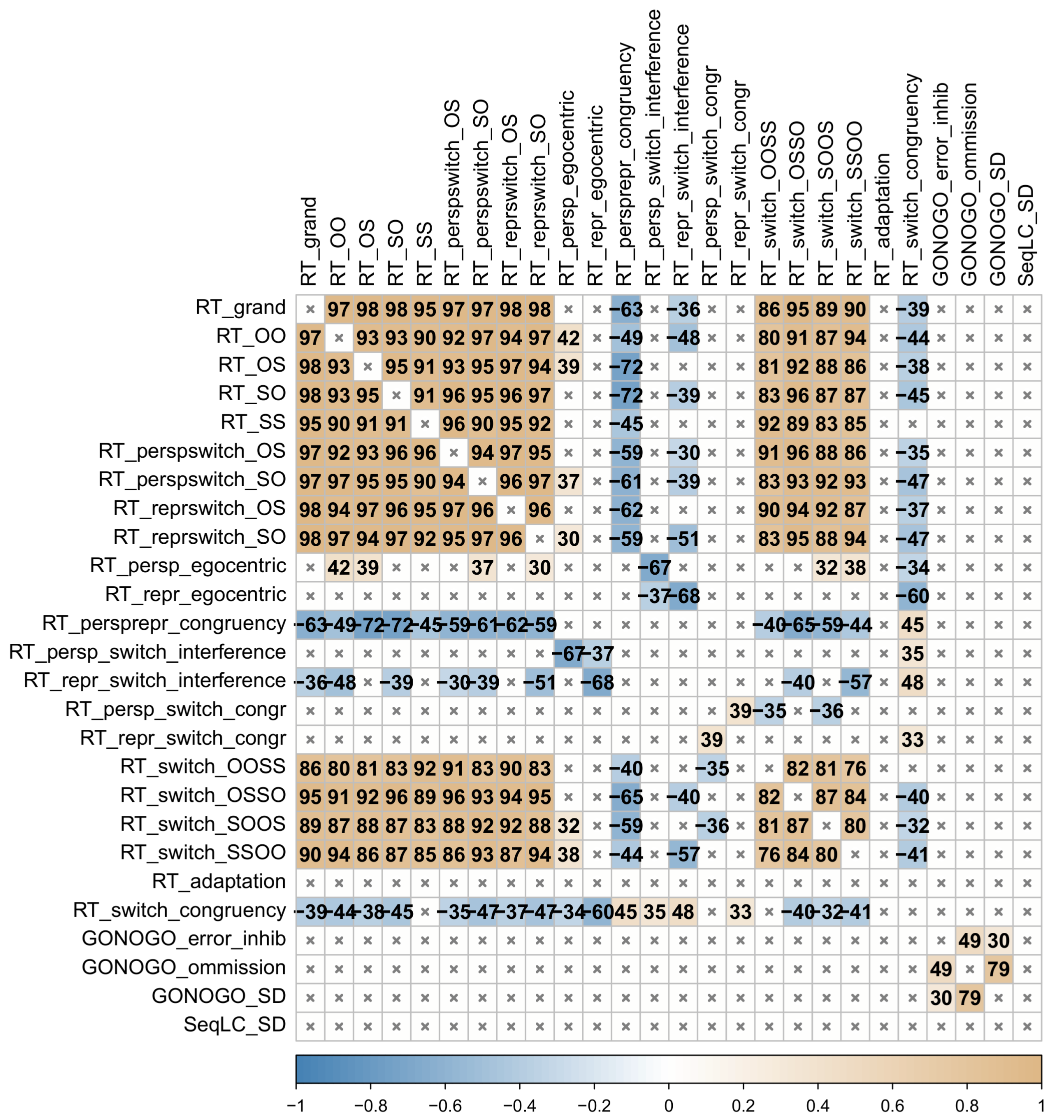

Correlations adults

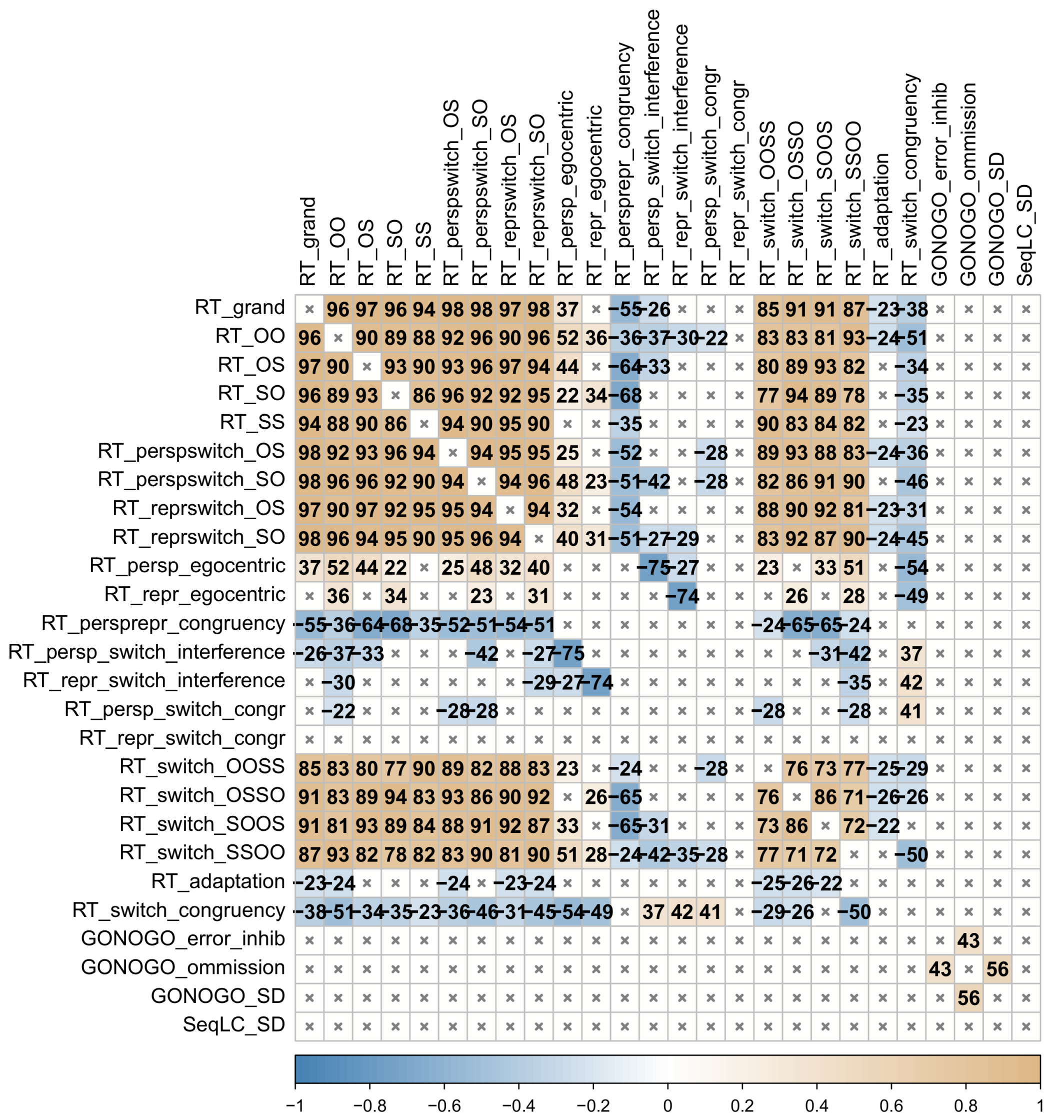

Supplement: sj-pdf-1-qjp-10.1177_17470218241311415 – Supplemental material for Unpacking mentalizing: The roles of age and executive functioning in self-other appraisal and perspective taking [file sj-pdf-1-qjp-10.1177_17470218241311415.pdf]
